# Update on the Molecular Pathology of Cutaneous Squamous Cell Carcinoma

**DOI:** 10.3390/ijms24076646

**Published:** 2023-04-02

**Authors:** Elena-Codruta Cozma, Laura Madalina Banciu, Cristina Soare, Sanda-Maria Cretoiu

**Affiliations:** 1 Dermatology Department, Elias University Emergency Hospital, 011461 Bucharest, Romania; codrutadobrica@gmail.com (E.-C.C.);; 2 Pathophysiology Department, University of Medicine and Pharmacy of Craiova, 200349 Craiova, Romania; 3 Surgery Department, Carol Davila University of Medicine and Pharmacy, 050474 Bucharest, Romania; 4 Department of Cell and Molecular Biology and Histology, Carol Davila University of Medicine and Pharmacy, 050474 Bucharest, Romania

**Keywords:** squamous cell carcinoma, skin cancer, cancer stem cells, epigenetics, targeted therapies

## Abstract

Cutaneous squamous cell carcinoma (cSCC) is the second most common skin cancer, originating from keratinocytes of the spinous layer. Numerous risk factors have been discovered for the initiation and growth of this type of cancer, such as exposure to UV and ionizing radiation, chemical carcinogens, the presence of immunosuppression states, chronic inflammation, infections with high-risk viral strains, and, last but not least, the presence of diseases associated with genetic alterations. The important socio-economic impact, as well as the difficulty associated with therapy for advanced forms, has made the molecular mechanisms underlying this neoplasia more and more intensively studied, with the intention of achieving a better understanding and advancing the treatment of this pathology. This review aims to provide a brief foray into the molecular, genetic, and epigenetic aspects of this cancer, as well as the treatment methods, ranging from the first used to the latest targeted therapies.

## 1. Introduction

**Figure 1:**
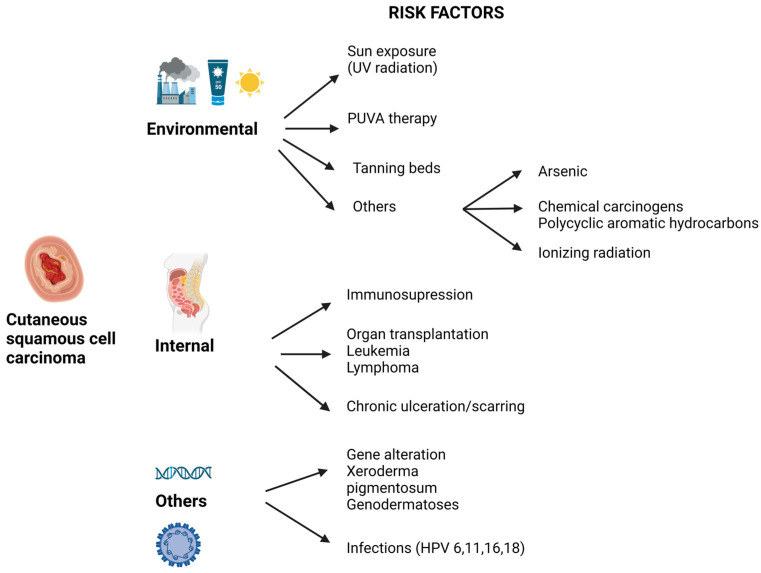
Risk factors for the development of cutaneous squamous cell carcinoma (This figure is created with BioRender.com, Agreement number NI24Y4NAV7) [3,4,5].

Squamous cell carcinoma (SCC) is one of the most common neoplasms (second place among all neoplasia), originating from keratinocytes in the spinous layer of keratinized stratified squamous epithelium [1]. This origin makes possible the occurrence of SCC types at the level of all organs and tissues that contain stratified squamous epithelia, such as the skin or mucous membranes lining the hollow organs (digestive tract, oral cavity, and respiratory tract epithelium). Regarding cutaneous SCC (cSCC), it ranks second among non-melanocytic skin cancers, after basal cell carcinoma, with an increasing incidence in recent years in Europe, although the incidence rate is stable in the USA and Australia [2,3]. Regarding risk factors, exposure to ultraviolet radiation (UVA and UVB) is the main cause of carcinogenesis in cSCC; by inducing DNA alterations in tumor suppressor genes or in pro-oncogenes, the risk increases through cumulative exposure throughout life. Other incriminated risk factors are represented by immunosuppression, chronic infections (especially with Human Papilloma Virus—HPV), genetic changes in genes involved in DNA repair, chronic ulceration, and chronic inflammation (Figure 1) [4,5].

The complex molecular mechanisms involved in the occurrence of cSCC, as well as the important mutational burden, translate into the presence of a large number of precursor forms of cSCC (actinic keratosis, Bowen’s disease, Queyrat’s erythroplasia, and Bownoid papulosis), as well as in situ or invasive cSCC (more than 15 different types reported in the literature from an anatomical–clinical point of view, including metatypical, verrucous, acantholytic, fusiform, pigmented, desmoplastic, mucoepidermoid, clear cell, signet ring cells, trichilemmal, inflammatory, lymphoepithelioma-like, basaloid, carcinosarcoma, papillary, and invasive Bowen’s disease) [4]. The World Health Organization has summarized these types into six forms: verrucous SCC, acantholytic SCC, lymphoepithelial SCC, clear cell SCC, spindle cell SCC, and SCC with sarcomatoid differentiation [6]. This review aims to highlight the main molecular mechanisms involved in carcinogenesis, as well as the epigenetic aspects that can influence treatment and treatment resistance.

## 2. Mechanisms of SCC Carcinogenesis: Molecular Pathogenesis of SCC

Most SCCs do not arise as de novo tumors, but in an incremental manner from premalignant or noninvasive precursor lesions [7].

Actinic keratosis (AK) represents in a clinical fashion the first detectable precursor lesion of cSCC. Most AKs can either remain in the premalignant status or even regress spontaneously [8]. A small subset of AKs acquire additional genetic and epigenetic changes and progress to cutaneous squamous cell carcinoma in situ (SCCIS) and furthermore to cSCC. The risk of evolution from AK to SCC is very difficult to predict, with the numbers varying vastly between different studies (0.025–20%) [9]. Out of the cSCCs, only a small percentage can acquire additional genetic and epigenetic features that lead to metastatic disease [10].

Ultraviolet (UV) exposure is considered a risk factor that initiates the mutagenic process in the skin, leading to modified keratinocytes that have a survival advantage over unmutated keratinocytes; this then leads to the risk of selection of mutated keratinocytes over time. These mutated clones can acquire further genetic or epigenetic changes, leading to AKs, and further to SCCISs and cSCCs. 

The development of cSCC is a multistep process requiring the accumulation of multiple genetic and epigenetic alterations in keratinocytes. These alterations lead to an augmented mutation rate by increasing cellular proliferation and reducing cell death in mutated keratinocyte population. DNA mutations are caused by either exogenous factors, such as UV radiation, chemicals, and ionizing radiation, or endogenous factors, such as reactive oxygen species (ROS), genome editing, mitotic errors, or errors in DNA repair [11,12]. 

Cumulative lifetime exposure to UV radiation is considered to be the most important carcinogen responsible for cSCC [13]. UV exposure over-activates the DNA repair systems of keratinocytes, leading to ATP consumption [14]. UVB radiation can produce DNA damage through structural rearrangements due to its high photonic energy. In case of a lack of repair of the damaged DNA strand before replication, the complementary strand will integrate the change in its base, leading to a constituted mutation [15]. This process leads to high rates of C > T transitions and CC > TT double base changes, thus generating a “UVB signature” [16,17]. Multiple genes have been postulated to be involved in the development of AKs and SCCs, with several molecular pathways and mechanisms being involved (Table 1).

## 3. Epigenetic Aspects of SCC: The Role of Epigenetics in Diagnosis, Metastatic Profile, and Prognosis

In recent years, special attention has been given to epigenetics and its involvement in the occurrence of chronic diseases in general, and cancers in particular [40]. Epigenetic changes include all the mechanisms through which changes occur in the expression of some genes, without interfering with the sequence of the nitrogenous bases that makes up the respective genes [41]. These are a result of the interactions between an organism and its environment, being represented by DNA methylation; histone modifications that influence the reading of certain DNA sequences; and miRNA-induced modifications, which can be transmitted from one cell to another within the same organism and even trans-generationally [42,43].

In general, the DNA of tumor cells is epigenetically characterized by global hypomethylation, with areas of hypermethylation at the level of 5’ cytosine-phosphate-guanine-3’ (CpG) islands, which are generally located in the promoter regions of some key genes. The above changes lead to genomic instability, activation of oncogenes, alteration of promoters of tumor suppressor genes, as well as damage to numerous essential cellular pathways involved in DNA repair, apoptosis, cell growth, angiogenesis, etc. [44,45,46]. 

In skin cancers, the involvement of epigenetics in the pathophysiology and characterization of melanoma is already recognized. These epigenetic mechanisms are considered as representing some of the earliest events in the initiation of oncogenesis [47]. However, the role of the interactions between the genome and the environment in the appearance and development of SCC, the second most common skin cancer, is less studied. The epigenetic profile seems to represent an important tool for characterizing the aggressiveness and metastatic potential of this type of skin cancer [48]. Moreover, multiple changes, such as CpG hypermethylation, seem to be involved in its occurrence [49]. The hypermethylation of certain CpG areas (induced especially by the effect of ultraviolet radiation, -thereby increasing the expression of dimethyltransferase 1) leads to changes in some proteins with an important role in keratinocyte homeostasis, which is associated with aggressive behavior and metastasis [50] (Table 2). Regarding post-translational modifications at the level of histones (through the processes of phosphorylation, acetylation, sumoylation, ubiquitylation, ribosylation of DNA, and glycosylation), these changes influence the way in which DNA sequences are exposed to reading, so that the transcription of some genes involved in keratinocyte differentiation is altered [51].

Besides DNA methylation, microRNA (miRNA) gene regulation is also present in the evolution of cSCC. Two types of miRNA are identified: those involved in the oncogenic process (which are involved in increasing cells’ proliferation and invasion capacity, the migration of keratinocytes, the formation of new cell colonies, and the loss of apoptotic capacity), and those with tumor suppressor capacity (which act by opposite mechanisms). MiR-203 is one of the most important tumor suppressor microRNAs involved in the pathogenesis of cSCC (being expressed in high levels in the skin), acting by modulating the expression of the oncogene *c-MYC* (suppressing its activity) and inhibiting the angiogenesis and cell cycle of tumoral cells. Additionally, a decrease in MiR-203 is associated with a low degree of cSCC differentiation and a worse prognosis [52]. Lohcharoenkal et al. have highlighted that MiR-130a also has tumor suppressor activity in cSCC by altering the bone morphogenetic protein (BMP)/SMAD pathway involved in tumor growth and invasion capacity. Thus, lower levels of MiR-130a have been found in cSCC samples compared to precancerous lesions or healthy skin [53]. Another miRNA that plays an important role in suppressing the proliferation and invasion of tumoral cells is miR-27; the downregulation of this MiRNA is associated not only with UVB radiation of the skin, but also with cSCC development [54]. MiRNAs 34a, 125b, 181a, 148a, 20a, 204, 199a, 124, and 214 are some of the investigated tumor suppressor MiRNAs involved in tumor progression, cell proliferation and differentiation, angiogenesis, and cell migration by targeting the expression of essential genes involved in these pathways [55]. Thus, lower expressions of these MiRNAs are observed in cSCC compared with normal skin. 

On the contrary, numerous MiRNAs has been identified to promote tumoral cell initiation and progression, acting as protooncogenes. MiR-221 is a microRNA involved in numerous cancers (gastric cancer, ovarian cancer, breast cancer, etc.), with recent studies showing an upregulation of this small RNA fragment in cSCC by suppressing phosphatase and tenesin homolog (PTEN) gene, a tumor suppressor gene [56]. Yin et al. identified another microRNA involved in cSCC, highlighting that MiR-21 is upregulated in cSCC tissues by being involved in the invasion and metastasis of cSCC and by decreasing the activity of (tissue inhibitor of matrix metalloproteinase 3) *TIMP3* gene. This gene is essential in modulating the activity of matrix metalloproteinases and molecules involved in angiogenesis, cell growth, and metastasis [56,57]. MiR-186 influences the aggressive character of cSCC, with its upregulation determining the inhibition of apoptotic protease-activating factor 1 (APAF 1) [58]. Additionally, some MiRNAs can be identified as prognosis factors. For example, a study conducted by Canueto et al. associated the presence of MiR-205 with a poor prognosis, being expressed in tumors characterized by histological risk factors, such as desmoplasia, nerve invasion, or an infiltrative character [59]. MiR 365, 31, 142, and 135b were also found to be involved in the regulation of genes responsible for cell invasion, migration, resistance to apoptosis, and proliferation [55]. Upregulation of MiR-664, 504, and 217 found in primary tumors seems to be associated also with the presence of an invasive behavior and a higher risk for metastatic disease. Gilespie et al. identified a group of miRNAs that are upregulated in tumors that metastasize compared to the primary one (miR-4286, miR-200a-3p, and miR-148-3p) and another group with aberrant expression in tumors with a high potential to metastasize (MiR-4286, MiR-421, MiR-4516, MiR-574-5p, MiR-135b, MiR-21, MiR-145, MiR-100, and MiR-214). Thus, these groups may be used in the future as markers of poor prognosis [60,61]. 

Regarding the role of histone changes in cSCC initiation and progression, the literature data are poor in identifying specific histone methylation and acetylation changes in sCC, even though their role in other cancers is well known. In cSCC, Enhancer Of Zeste 2 Polycomb Repressive Complex 2 Subunit (*EZH2*) (involved in histone methylation) seems to play a role in inhibiting the antitumoral immune response of the host, and it can be used in the future as an important target of specific antitumoral therapy [62]. 

**Table 2:** DNA methylation changes associated with cSCC.

Epigenetic Changes	Involved Gene	Involved Protein	Role of the Protein	Results
DNA methylation of CpG, leading to loss of function	*CDH1* gene promoter (E-cadherin gene promoter)	E-cadherin	Intercellular adhesion molecule	Hypermethylation of this gene is more frequent in invasive forms of SCC compared to AK and normal population. Hypermethylation of *CDH1* gene promoter is present in 95% of cutaneous squamous cell carcinoma samples [63,64].
DNA methylation of CpG, leading to loss of function	*CDH13* gene promoter (T-cadherin gene promoter)	T-cadherin	Cell migration; Phenotypic changes; Calcium ion transport	Hypermethylation of T-cadherin gene promoter is associated with phenotypic cellular changes, both in SCC and in actinic keratoses that will evolve into invasive SCC [65,66].
DNA methylation of CpG, leading to loss of function	*CDKN2A* gene	p16 (ARF)	Tumor suppressor genes—cell cycle regulator proteins	Hypermethylation is associated with a statistically significant decrease in the synthesis of aforementioned proteins (identified by immunohistochemical studies) (*p* < 0.001), with the inactivation of p16 and p14 [64].
DNA methylation of CpG, leading to loss of function	*CDKN2A* gene	p14 (INK4A)		
DNA methylation of CpG, leading to loss of function	Retinoblastoma protein 1 (RB1) gene	*RB1*		
DNA methylation of CpG, leading to loss of function	*SFRP1-5* promoter gene	SFRP1-5 glycoproteins	Modulatory effects on Wnt pathway	Hypermethylation of the promoter region of these genes leads to a decrease in the expression of SFRP1-5 proteins in cSCC. It can be used as a marker of cSCC [67].
DNA methylation of CpG, leading to loss of function	*FRZB* (frizzled-related protein) promoter gene	FRZB protein	Modulatory effects on Wnt pathway—involved in cell growth and differentiation	Hypermethylation of the promoter of this gene is significantly higher in metastatic cSCC compared to non-metastatic forms (*p* = 0.00004). It can be used as a marker of subtypes with aggressive evolution [68].
DNA methylation of CpG, leading to loss of function	Death-associated protein kinase 1 (DAPK1) gene	*DAPK1*	Tumor suppressor activity—protein involved in apoptosis and autophagy	*DAPK1* hypermethylation is much more frequent in invasive forms of cSCC [66].

## 4. Tumor Heterogeneity: Cancer Stem Cells and Therapy Resistance

The understanding of cancer biology was revolutionized by the discovery of cancer stem cells (CSCs) by Bonnet and Dick, who described these cells in human acute myeloid leukemia [69]. Nowadays, the molecular events happening in the microenvironment, involving also stromal cells of non-tumoral nature, and the tumoral–epithelial cell interactions are just beginning to be deciphered, but they seem to play a significant role in understanding tumor progression and resistance to therapy. In a tumor, it seems that not all cells are equal, but only a small proportion possesses the capacity of self-renewal and hierarchical differentiation, and these are the CSCs [70]. There are several cell types that are considered as contributing to the heterogeneity: tumor cells, non-stem cancer cells, CSCs, cancer-associated fibroblasts (CAFs), endothelial cells, pericytes, tumor-associated macrophages (TAMs), mesenchymal stem cells (MSCs), and MSC-derived cells [71]. Additionally, one must keep in mind that tissue stem cells, cells of origin (tumor-initiating cells), and CSCs are distinct concepts [72]. Tumor-initiating cells are those subjected to the initial mutation and will develop to form the tumor detected by clinical means, while CSCs are cells responsible for the response to clinical treatments, drug resistance, tumor relapse, and propagation at distance (metastasis) [73].

CSCs are considered to be the basis of any tumor development and responsible for intratumoral genetic and phenotypic heterogeneity [74,75]. Like normal stem cells, CSCs reside in a specific microenvironment similar to stem cell niche, called a CSC niche [76]. Moreover, CSCs are also responsible for repeating the phenotypic features of primary tumors in secondary tumors and for drug resistance [77]. For example, in SCC, there are multiple population of CSCs with different phenotypes, including those responsible for tumorigenesis, proliferation, and tumor growth, and others responsible for the epithelial-mesenchymal transition and metastatic processes [78]. Moreover, there are subpopulations of CSCs that remain in a dormant state, which makes them difficult to be targeted by drugs that affect the cell cycle, and are at the origin of drug resistance and relapse after chemotherapy [78]. It is well known that cancer cells can suffer to show plasticity under the influence of microenvironmental factors (stromal cells, extracellular matrix molecules, and systemic and local growth factors), becoming CSCs that are able to transdifferentiate and dedifferentiate [79]. In the view of recent discoveries, plasticity can be subdivided into extrinsic plasticity determined by changes in the microenvironment and intrinsic plasticity induced by specific transcription factors [80]. In fact, based on current data, there are three general models describing tumor heterogeneity: (1) the clonal evolution (CE) model; (2) the cancer stem cell (CSC) model; and (3) the plasticity model (for details, see review [81]). The CE model is based on the theory of Darwinian evolution in which a single cell undergoes mutations that are then transmitted through division to the daughter cells. The most adaptable daughter cells will survive and the others will disappear as a result of natural selection [82]. The CSC model is based on the existence of a small group of cells inside tumors that have stem cell traits and the potential to proliferate hierarchically, thus being responsible for the induction, propagation, and metastasis of tumors [83]. The plasticity model is based on the ability of CSCs and non-CSCs to shift states among each other [80]. Although the role of the CE model and the plasticity model are not yet highlighted in the case of cSCC, the role of CSCs in the initiation and perpetuation of non-melanocytic skin cancers (especially SCC) is well known; the number of CSCs in a tumor varies between 1–20%, with the percentage increasing with the aggressiveness of SCCs. Moreover, the types of CSCs involved in the appearance of SCCs can be accurately identified by highlighting specific cellular markers on the surface of these cells, such as CD34, CD200, and CD 44 [78]. Regarding cancer cell plasticity, epigenetic changes seem to be involved in the regulation of tumor microenvironment, which influences the continuous transition of tumor cells from stem to non-stem cancer cells, from an active to a quiescent state, and from an epithelial to a mesenchymal status. This behavior is well known in the case of epithelial tumors, but it is insufficiently investigated in the specific case of cSCC [84,85].

A lot of intrinsic and extrinsic factors are responsible for regulating the stemness in CSCs, as described in the following subsections. 

## 5. Novel Therapeutic Approaches in Cutaneous SCC (Targeted Therapies and Immunotherapies)

### 5.1. Surgery

Regarding treatment, the main objective is a complete removal of the tumor, along with the maximum preservation of healthy surrounding tissues and good cosmetic results. Classic early surgical excision is the treatment of choice for localized stages, with a cure rate of >90% at five years [86].

According to the EDF-EADO-EORTC group, the limits of surgical resection are 5 mm margins for low-risk tumors and extended up to 10 mm for high-risk tumors [87].

Mohs microsurgery with margin control may be an option in patients with high risk and/or with special anatomical locations, given the increased curability associated with minimal recurrence rates, maximum tissue preservation, and good esthetic results [88].

A percentage of 4–5% of patients with SCC progress to more advanced stages: advanced locally, respectively metastatic diseases (<5%) with locoregional or distant metastases; those stages require other therapeutic approaches, such as chemotherapy, radiotherapy, or more recently immunotherapy. The low incidence of metastatic forms makes these forms a therapeutic challenge; the management of these patients must be based on the medical decisions of a multidisciplinary team of dermatologists, surgeons, radiotherapists, and oncologists [89].

The staging of cSCC is performed according to the criteria established by the AJCC 8th edition Staging Manual (American Joint Committee on Cancer, 2017) and the UICC 8th edition (Union for International Cancer Control, 2017). Stratification according to risk is carried out according to the characteristics related to tumor or patient. According to the EADO guide for the diagnosis and treatment of cSCC, low-risk tumors are pT1 tumors (tumor <2 cm in its greatest dimension according to (AJCC8)) or tumors that do not present the risk factors established by the EADO. High-risk tumors are those with at least a pT2 stage (tumor larger than 2 cm) (AJCC8) or those that are associated with the EADO risk factors. However, the exact impact of each risk factor on recurrence is not known [90]. Current treatment guidelines (AJCC 8th ed. classification, BWH classification of the Brigham Women’s Hospital, NCCN Guidelines, and EADO guidelines) attempt to systematize these risk factors in order to be able to classify patients’ stage of disease, with subsequent impact on the choice of treatment. The risk factors related to patients are immunosuppression, appearance of carcinoma in a radio-treated area or with chronic inflammation, and symptoms indicating perineural invasion. The risk factors related to tumor are diameter greater than 2 cm, location of the tumor in a high-risk area, imprecise delimited edges, rapid tumor growth, and recurrence. Radiological risk factors include bone invasion and perineural invasion. Histological risk factors include tumor thickness >6 mm, poor differentiation, high-risk histological subtypes, perineural invasion, lymphatic/vascular invasion, and subcutaneous tissue invasion [91]. 

The main role of these classification systems is to choose an appropriate management for each patient with cSCC. Thus, cSCC is divided into primary cSCC and metastatic cSCC. Primary cSCC can be primary low-risk in which the treatment of choice is excision with 5 mm margins or primary high-risk in which the curative solution is excision with 6–10 mm oncological margins or Mohs micrographic surgery. Locally advanced primary cSCC and metastatic cSCC (metastases in transit, nodal metastases, or distant metastases) require a multidisciplinary and individualized approach for each patient because negative margins cannot be obtained through the surgical approach. In addition to classical therapies, such as radiotherapy, electrochemotherapy, adjuvant radiotherapy, and chemotherapy, with the understanding of tumor molecular mechanisms, new classes of drugs have been introduced, such as immunotherapy with growth factor inhibitors or combined treatments [87]. 

### 5.2. Radiation Therapy

Radiation therapy can be used as a therapeutic option for in situ SCC in patients over 60 years of age, with multiple lesions located on the lips, or those refusing therapy, but it has a higher risk of recurrence than classic excision. It can also be an adjuvant therapy in patients with more advanced stages. For locally advanced SCC, radiotherapy can be used in case of perineural invasion or as an adjuvant method in case of positive post-excision margins. Side effects include mucositis/dermatitis; telangiectasia; hypodermic sclerosis; necrosis of the soft tissue, cartilage, and bone; decreased sensitivity; and skin carcinomas. However, the high risk of recurrence compared to complete surgical excision should be considered [92].

### 5.3. Systemic Therapy

Systemic therapy is a therapeutic option in patients with locally advanced SCC and/or metastases despite previous therapies.

In 2020, European interdisciplinary guidelines (EADO, EDF, and EORTC) have created a number of high-risk prognostic factors for cSCC recurrence, such as clinical features (location, symptomatic perineural invasion, and tumor size), histological features (poor differentiation, desmoplasia, thickness, and perineural invasion), immunosuppression, and radiological features (bone erosion and radiological PNI), leading to the need for therapeutic protocols in these patients [90]. 

#### 5.3.1. Chemotherapy

In advanced cSCC, systemic therapies with cytotoxic agents have been used off-label: cisplatin/carboplatin, 5-fluorouracil, bleomycin, methotrexate, taxanes, gemcitabine, and polychemotherapy are proven more effective than monotherapy, but they are associated with more severe adverse reactions. More than three decades ago, the therapies used were isotretinoin, interferon, and cytotoxic agents, which showed efficacy on cSCC but were limited in effect on metastases [93].

Prior to the era of targeted therapy, platinum-based chemotherapies were the first line of treatment, but they were burdened by high toxicity and an increased risk of recurrence of the disease under treatment [94].

#### 5.3.2. Targeted Therapy

Due to recent progress made in the molecular biology of tumors, new targeted systemic therapies have been discovered to increase survival in advanced stages. Thus, cSCC is characterized by a high mutational tumor load with antigen formation, which can be targeted by the immune system. The role of the immune system in the pathogenesis of cSCC has been studied by observing the increased rate of cSCC in transplant patients or by the rapid involution of keratoacanthomas as a result of an active immune response [94].

Immunomodulators can be used in the treatment of cSCC due to the ability of the immune system to control the carcinogenesis process. The pathogenesis of cSCC is based on keratinocyte mutation with subsequent tumor clonal expansion under the action of exogenous and endogenous factors, such as immune suppression. In this context, the important cellular feature is self-tolerance mediated by surface expression of receptors and molecules known as immune checkpoints [89].

In cSCC, there is an excessive expression of these molecules, especially programmed cell death protein 1 (PD-1), programmed cell death ligand 1 (PD-L1), cytotoxic T-lymphocyte-associated antigen 4 (CTLA-4), and epidermal growth factor receptor (EGFR), which are molecules that can be therapeutically targeted [95]. In addition, there are numerous mechanisms of escape from immune surveillance through various cytokines, including increased secretion of IL-6, IL-10, and TGF-beta; decreased secretion of IL-2; and inhibiting the proliferation of CD4 + and CD8 + T lymphocytes with a role in the recognition of tumor antigens. At present, anti-programmed cell death-1 (anti-PD-1) antibodies are the first line of treatment for advanced metastatic/local cSCC that cannot be cured by local surgery or radiation [92].

After their activation, T lymphocytes express on the surface PD-1 molecules, with a role in the apoptosis of effector T cells and inhibition of T reg cell apoptosis by binding to PD-1 and PD-L2 in tumor cells. Tumor cells may overexpress PD-L1 by escaping under immune surveillance, which is associated with metastatic and recurrent cSCC and T cell exhaustion following chronic exposure to tumor antigens [89]. 

Co-inhibitory molecules play an important role in preventing hyperstimulation and autoimmunity. Programmed cell death-1 acts as a co-inhibitory receptor because it binds to T cells by binding to the PD-L1 ligand expressed in tumor cells, thereby preventing T cell activation and immunological exhaustion. This process is called immunosurveillance [96].

PD-L1 is expressed in 3–50% of cSCC, correlating with an increased risk of metastases [94].

The data from the literature show that PD-L1 expression is associated with high-risk SCC: infiltrative patterns, immunosuppression, and perineural invasion [97].

Currently, given that 50% of tumors do not respond to immunotherapy, attention is focused on identifying predictive factors for response, including tumoral genes and biomarkers in the peripheral blood. Thus, the tumor markers used may be PD-L1 status, IFN gamma expression, and tumor-infiltrating lymphocytes (TILs). Liquid biopsy markers can be immunophenotypic profile, cytokines and chemokines (IL-6), and soluble markers (sCTLA4 and sPD-L1) [96].

##### Anti-PD-1 Agents

- Cemiplimab

Cemiplimab is the first systemic therapy evaluated in prospective studies in patients with advanced cSCC. Approved for use in advanced cSCC therapy among patients who are not candidates for surgery or radiation therapy in September 2018 by the Food and Drug Administration (FDA) and in July 2019 by the European Medicines Agency (EMA), Cemiplimab is a humanized monoclonal antibody IgG4 with an affinity for PD-1. At doses of 350 mg iv every three weeks, it blocks the interaction of PD-1 with PD-L1 at the tumor level, thus restoring T-cell activity and antitumor response [98].

The data from the literature demonstrate efficacy (response rates of up to 46.1%) and sustained response while maintaining disease control in approximately 72% of patients with advanced SCC treated with Cemiplimab [88].

The efficacy of Cemiplimab has been tested in several phase I and phase II studies. In a phase I study with patients with locally advanced or metastatic disease led by M.R. Migden et al. (2018), a response rate of 50% was obtained, while in the cohort with metastatic disease (phase 2 study), a response rate of 47% was obtained. Of these patients, 7% had a response lasting more than six months, and side effects were reported in 15% of them [99].

Cemiplimab has a good safety profile. The most common side effects reported are diarrhea, fatigue, constipation, and rash, which can be resolved by adjusting treatment doses or sometimes stopping treatment, but the therapeutic benefits and long-term response outweigh the risks of side effects [98]. Cemiplimab has recently been included in clinical trials as adjuvant, neoadjuvant, or combined adjuvant/neoadjuvant therapy in patients with resectable or partially resectable SCC [86].

- Nivolumab

Nivolumab is a PD-1 inhibitor approved by the FDA in November 2016 for the treatment of head and neck SCC, following a study that enrolled 361 patients receiving Nivolumab at a dosage of 3 mg/kg every two weeks, with improved overall survival [100].

- Pembrolizumab

There are ongoing studies on the effectiveness of Pembrolizumab on cSCC. The interim results of the Keynote 629 study with a mean follow-up of 9.5 months, in which 200 mg/3 weeks of Pembrolizumab was used, showed a 32% response rate in 91 patients using it as the second line of treatment and a 50% response rate in 14 naive patients, but the average duration of response was unknown [101]. 

##### Anti-CTLA-4 Antibody

Regarding Ipilimumab, Day et al. reported a case of metastatic cSCC in a melanoma patient who received Ipilimumab every three weeks and completed three cycles. The patient responded to the therapy with decreasing cSCC metastases after three cycles of treatment, obtaining a partial response without significant adverse drug reactions [102]. 

##### Anti CTLA-4 Antibodies Combined with PD-1 Antibodies

Therapies can be successfully combined but at the cost of increasing side effects. Miller et al. reported the case of a 68-year-old patient who developed metastatic cSCC three years after kidney transplantation for which he received combination therapy with Ipilimumab and nivolumab with therapeutic response. The patient soon developed kidney failure, the transplant was removed, and he died a few months later due to cardiopulmonary arrest, which could not be attributed to the therapy [103].

##### Epidermal Growth Factor Receptor Inhibitors (iEGFR)

EGFR is a family of proteins that the human epidermal growth factor (HER) belongs, which activation determines the activation of multiple signaling pathways, including mitogen-activated protein kinase/Extracellular signal-regulated kinase ½ (MAPK/ERK) and phosphatidylinositol-3-kinase/protein kinase B/mammalian target of rapamycin(PI3K/AKT/mTOR), and plays a role in maturation, proliferation, inhibition of apoptosis even at the tumor level, leading to tumor growth. Regarding skin cancers, EGFR mutations have a low incidence of 2.5–5%, but are associated with the risk of metastases and, therefore, with a worse prognosis, thus becoming a therapeutic target. This has led to the discovery of anti-EGFR agents, cetuximab or panitumumab monoclonal antibodies, that competitively inhibit EGF receptors, or small molecules that target intracellular domains, including gefitinib or erlotinib [89].

- Cetuximab

Cetuximab is a chimeric immunoglobulin that binds to the extracellular domain 3 of EGFR. Thus, it determines an adaptive and innate immune response by downregulating the immunosuppressive mechanisms and decreasing PD-L1 (programmed death ligand 1) induced by IFN-gamma. By modulating the PD-1 axis, treatment with Cetuximab may lead to a decrease in the therapeutic effect of immunotherapies (PD-1 inhibitors) in patients with recurrent cSCC [104].

Cetuximab has been included in various studies to show its effectiveness. In a phase 3 study in France that included 36 patients with metastatic or advanced cSCC, locoregional response to the treatment was 28%, with a mean duration of response of seven months. Another phase 2 study in which cetuximab was used as a monotherapy in the treatment of unresectable cSCC found stabilization of the disease in 58% of cases. [105]. Commonly reported side effects were infections, tumor bleeding, infusion-related reactions, interstitial pneumonia. However, more studies are needed to examine the effectiveness of anti-EGFR and the possibility of combining them with other therapies [94]. 

### 5.4. Novel Approaches 

#### 5.4.1. Radiotherapy Associated with Immunotherapy

A new approach in cSCC management is the combination of radiation therapy and immunotherapy. Radiation causes damage to both the tumor and the surrounding normal tissues by stimulating the immune system. Thus, irradiation causes MHC-1 expression in tumor cells, triggering the recruitment of effector immune cells, with some, even with specific antitumor responses, acting synergistically with checkpoint inhibitors [95].

#### 5.4.2. Oncolytic Viruses

Oncolytic viruses targeting tumor cells cause less immune-tolerant tumor microenvironment, thereby causing subsequent cytokine expression, which acts synergistically with checkpoint inhibitors by increasing tumor CD8+ and interferon gamma (IFN-gamma) signaling and up-regulating PD-L1 in the tumor microenvironment. Such compounds include RP1 (Replimune -1), a modified herpes simplex 1, which can induce tumor regression by stimulating GALV-GP R-protein (glycoprotein of gibbon ape leukemia virus) and GM-CSF (granulocyte/macrophage colony-stimulating factor) when used alone or in combination with nivolumab. Another oncolytic virus is Talimogene laherparepvec (TVEC), a non-neurovirulating herpesvirus simplex capable of inducing GM-CSF [106,107]. 

### 5.5. Transplant Recipients

Transplant patients, due to prolonged immunosuppression, have a higher risk of cSCC with a much more aggressive character and a much higher risk of metastases. In these patients, a benefit of the switch from immunosuppressive therapy to sirolimus was observed, with minimal effects on the graft and no negative effects on patient survival. This is due to the observation that immunotherapies should be used with caution because anti-PD1 agents can cause irreversible allograft rejection, and anti-CTLA-4s have been shown to be better tolerated [94].

## 6. Conclusions

Advanced squamous cell carcinomas are a challenge for clinicians, and even with a multidisciplinary approach, they remain difficult to treat. Often, advanced tumors do not respond to classic treatment options, so new approaches are needed, but equally important are the prevention and detection of early stage tumors that can lead to an excellent prognosis. Most new therapies are still in clinical trials or need to be approved, but their therapeutic benefit is certain. Thus, for a better understanding of the pathophysiology of cSCC, the molecular, genetic, and epigenetic mechanisms behind the behaviors of tumor cells is the key to new targeted therapies, with minimal side effects for patients.

## Figures and Tables

**Table 1:** Mutated genes involved in the development of AKs and SCCs.

Gene	Role
*TP53*	The most involved mutated gene in all types of cancers, but also in SCCs [18]. *TP53* gene encodes p53 protein which plays a vital role in cell cycle. Even though mutations in *TP53* gene appears early in the evolution of SCCs, they cannot represent the sole mutation needed for cancerous progression [19].
*NOTCH*	Loss of function of *NOTCH* gene represents a premature sign of the conversion of healthy keratinocytes to their precancerous version [12,20]. Mutation of *NOTCH* genes has been found in both SCCs and dermoscopy-free dysplastic lesions that has been chronically exposed to sun, thus suggesting that *NOTCH* mutation can be considered a trademark for UV-damaged keratinocytes [21,22].
*RAS*	*RAS* mutation is characteristic for cSCCs developed in patients treated with Vemurafenib, a B-Raf inhibitor [23]. It is a rare spontaneous mutation in cSCCs [20,24].
*CDKN2A*	It encodes p16^INK4a^ and p14^ARF^, which have the role of tumor-suppressor proteins [25]. p16^INK4a^ inhibits cyclin D-dependent kinases in a direct manner, leading to the activation of retinoblastoma protein (RB) [26]. p14^ARF^ isolates Mdm2 inside the nucleolus, preventing its interaction with p53 [27,28]. Mutation of *CDKN2A* plays an important role in both the appearance of AKs and the evolution of AKs to SCC [29]
*PIK3CA*	*PIK3CA* mutations were found in almost half of the cohort in a study, including AKs and SCCs, while in another cohort involving head and neck SCCs, ⅓ was found [24]. Tirbanibulin 1% ointment is used in the topical treatment of AKs with good efficacy, being based on a Src kinase inhibitor that acts on inhibiting PI3K (phosphatidylinositol 3-kinase) [30].
*KNSTRN*	It is considered to be part of the “UVB signature” [17]. It encodes a kinetochore protein that leads to the disruption of chromatin cohesion and, thus, aneuploidy and tumorigenesis [24].
*FAT1*	It encodes a protocadherin that can lead either to aberrant Wnt/β-catenin signaling or to increased CDK6 expression [31].
*CARD11*	*CARD11* gene encodes a protein that scaffolds the role of nuclear factor kappa B (NF-kB) [32]. *CARD11* mutations are found in UV-exposed skin and in the skin surrounding cSCC, and they have a role in the progression from normal to cancerous keratinocytes [33].
*SRCASM*	It encodes a tumor suppressor molecule with a VHS site, a GAT site, and multiple tyrosine phosphorylation domains [34]. Src-family tyrosine kinase (SFK) phosphorylation of the above molecule leads to limitation of keratinocyte proliferation and engagement in keratinocyte differentiation [34]. Both SCCISs and SCCs are associated with reduced *SRCASM* levels and a raised activity of SFKs [35,36].
*TP63*	*TP63* gene encodes p63 protein, which is commonly found to be overexpressed in cSCCs [37].
*EGFR*	*EGFR* is found to be dysregulated in the development of cSCCs [38]. *EGFR* is involved in keratinocyte proliferation and differentiation and also influences the rate of survival of these cells [39].

## References

[1] Sanchez-Danes A., Blanpain C. (2018). Deciphering the cells of origin of squamous cell carcinomas. Nat. Rev. Cancer.

[2] Stang A., Khil L., Kajuter H., Pandeya N., Schmults C.D., Ruiz E.S., Karia P.S., Green A.C. (2019). Incidence and mortality for cutaneous squamous cell carcinoma: Comparison across three continents. J. Eur. Acad. Dermatol. Venereol..

[3] Que S.K.T., Zwald F.O., Schmults C.D. (2018). Cutaneous squamous cell carcinoma: Incidence, risk factors, diagnosis, and staging. J. Am. Acad. Dermatol..

[4] Bonerandi J.J., Beauvillain C., Caquant L., Chassagne J.F., Chaussade V., Clavere P., Desouches C., Garnier F., Grolleau J.L., Grossin M. (2011). Guidelines for the diagnosis and treatment of cutaneous squamous cell carcinoma and precursor lesions. J. Eur. Acad. Dermatol. Venereol..

[5] Kim Y., He Y.Y. (2014). Ultraviolet radiation-induced non-melanoma skin cancer: Regulation of DNA damage repair and inflammation. Genes Dis..

[6] International Agency for Research on Cancer WHO Classification of Tumour. https://tumourclassification.iarc.who.int/welcome/#preview.

[7] Criscione V.D., Weinstock M.A., Naylor M.F., Luque C., Eide M.J., Bingham S.F., Department of Veteran Affairs Topical Tretinoin Chemoprevention Trial Group (2009). Actinic keratoses: Natural history and risk of malignant transformation in the Veterans Affairs Topical Tretinoin Chemoprevention Trial. Cancer.

[8] Sartini D., Campagna R., Lucarini G., Pompei V., Salvolini E., Mattioli-Belmonte M., Molinelli E., Brisigotti V., Campanati A., Bacchetti T. (2021). Differential immunohistochemical expression of paraoxonase-2 in actinic keratosis and squamous cell carcinoma. Hum. Cell.

[9] Righi V., Reggiani C., Tarentini E., Mucci A., Paganelli A., Cesinaro A.M., Mataca E., Kaleci S., Ferrari B., Meleti M. (2021). Metabolomic Analysis of Actinic Keratosis and SCC Suggests a Grade-Independent Model of Squamous Cancerization. Cancers.

[10] Ratushny V., Gober M.D., Hick R., Ridky T.W., Seykora J.T. (2012). From keratinocyte to cancer: The pathogenesis and modeling of cutaneous squamous cell carcinoma. J. Clin. Investig..

[11] Friedberg E.C., Wood R.D., Walker G.C., Schultz R.A., Siede W., Ellenberger T., ProQuest (2006). DNA Repair and Mutagenesis.

[12] Martincorena I., Campbell P.J. (2015). Somatic mutation in cancer and normal cells. Science.

[13] Inman G.J., Wang J., Nagano A., Alexandrov L.B., Purdie K.J., Taylor R.G., Sherwood V., Thomson J., Hogan S., Spender L.C. (2018). The genomic landscape of cutaneous SCC reveals drivers and a novel azathioprine associated mutational signature. Nat. Commun..

[14] Campagna R., Pozzi V., Sartini D., Salvolini E., Brisigotti V., Molinelli E., Campanati A., Offidani A., Emanuelli M. (2021). Beyond Nicotinamide Metabolism: Potential Role of Nicotinamide N-Methyltransferase as a Biomarker in Skin Cancers. Cancers.

[15] Mitchell D.L., Nairn R.S. (1989). The biology of the (6–4) photoproduct. Photochem. Photobiol..

[16] Brash D.E., Rudolph J.A., Simon J.A., Lin A., McKenna G.J., Baden H.P., Halperin A.J., Ponten J. (1991). A role for sunlight in skin cancer: UV-induced p53 mutations in squamous cell carcinoma. Proc. Natl. Acad. Sci. USA.

[17] Brash D.E. (2015). UV signature mutations. Photochem. Photobiol..

[18] Martincorena I., Roshan A., Gerstung M., Ellis P., Van Loo P., McLaren S., Wedge D.C., Fullam A., Alexandrov L.B., Tubio J.M. (2015). Tumor evolution. High burden and pervasive positive selection of somatic mutations in normal human skin. Science.

[19] Hedberg M., Seykora J.T. (2021). Clarifying Progress on the Genomic Landscape of Actinic Keratosis. J. Investig. Dermatol..

[20] Thomson J., Bewicke-Copley F., Anene C.A., Gulati A., Nagano A., Purdie K., Inman G.J., Proby C.M., Leigh I.M., Harwood C.A. (2021). The Genomic Landscape of Actinic Keratosis. J. Investig. Dermatol..

[21] Chitsazzadeh V., Coarfa C., Drummond J.A., Nguyen T., Joseph A., Chilukuri S., Charpiot E., Adelmann C.H., Ching G., Nguyen T.N. (2016). Cross-species identification of genomic drivers of squamous cell carcinoma development across preneoplastic intermediates. Nat. Commun..

[22] Quintana R.M., Dupuy A.J., Bravo A., Casanova M.L., Alameda J.P., Page A., Sanchez-Viera M., Ramirez A., Navarro M. (2013). A transposon-based analysis of gene mutations related to skin cancer development. J. Investig. Dermatol..

[23] Ashford B.G., Clark J., Gupta R., Iyer N.G., Yu B., Ranson M. (2017). Reviewing the genetic alterations in high-risk cutaneous squamous cell carcinoma: A search for prognostic markers and therapeutic targets. Head Neck.

[24] Ji A.L., Rubin A.J., Thrane K., Jiang S., Reynolds D.L., Meyers R.M., Guo M.G., George B.M., Mollbrink A., Bergenstrahle J. (2020). Multimodal Analysis of Composition and Spatial Architecture in Human Squamous Cell Carcinoma. Cell.

[25] Meek D.W., Anderson C.W. (2009). Posttranslational modification of p53: Cooperative integrators of function. Cold Spring Harb. Perspect. Biol..

[26] Zilfou J.T., Lowe S.W. (2009). Tumor suppressive functions of p53. Cold Spring Harb. Perspect. Biol..

[27] Sherr C.J. (2006). Divorcing ARF and p53: An unsettled case. Nat. Rev. Cancer.

[28] Honda R., Yasuda H. (1999). Association of p19(ARF) with Mdm2 inhibits ubiquitin ligase activity of Mdm2 for tumor suppressor p53. EMBO J..

[29] Al-Rohil R.N., Tarasen A.J., Carlson J.A., Wang K., Johnson A., Yelensky R., Lipson D., Elvin J.A., Vergilio J.A., Ali S.M. (2016). Evaluation of 122 advanced-stage cutaneous squamous cell carcinomas by comprehensive genomic profiling opens the door for new routes to targeted therapies. Cancer.

[30] Heppt M.V., Dykukha I., Graziadio S., Salido-Vallejo R., Chapman-Rounds M., Edwards M. (2022). Comparative Efficacy and Safety of Tirbanibulin for Actinic Keratosis of the Face and Scalp in Europe: A Systematic Review and Network Meta-Analysis of Randomized Controlled Trials. J. Clin. Med..

[31] Morris L.G., Kaufman A.M., Gong Y., Ramaswami D., Walsh L.A., Turcan S., Eng S., Kannan K., Zou Y., Peng L. (2013). Recurrent somatic mutation of FAT1 in multiple human cancers leads to aberrant Wnt activation. Nat. Genet..

[32] Chalmers Z.R., Connelly C.F., Fabrizio D., Gay L., Ali S.M., Ennis R., Schrock A., Campbell B., Shlien A., Chmielecki J. (2017). Analysis of 100,000 human cancer genomes reveals the landscape of tumor mutational burden. Genome Med..

[33] Watt S.A., Purdie K.J., den Breems N.Y., Dimon M., Arron S.T., McHugh A.T., Xue D.J., Dayal J.H., Proby C.M., Harwood C.A. (2015). Novel CARD11 Mutations in Human Cutaneous Squamous Cell Carcinoma Lead to Aberrant NF-kappaB Regulation. Am. J. Pathol..

[34] Seykora J.T., Mei L., Dotto G.P., Stein P.L. (2002). ‘Srcasm: A novel Src activating and signaling molecule. J. Biol. Chem..

[35] Li W., Marshall C., Mei L., Dzubow L., Schmults C., Dans M., Seykora J. (2005). Srcasm modulates EGF and Src-kinase signaling in keratinocytes. J. Biol. Chem..

[36] Li W., Marshall C., Mei L., Gelfand J., Seykora J.T. (2007). Srcasm corrects Fyn-induced epidermal hyperplasia by kinase down-regulation. J. Biol. Chem..

[37] Malanchi I., Peinado H., Kassen D., Hussenet T., Metzger D., Chambon P., Huber M., Hohl D., Cano A., Birchmeier W. (2008). Cutaneous cancer stem cell maintenance is dependent on beta-catenin signalling. Nature.

[38] Wang Y., Deng X., Dai Y., Niu X., Zhou M. (2019). miR-27a Downregulation Promotes Cutaneous Squamous Cell Carcinoma Progression via Targeting EGFR. Front. Oncol..

[39] Jost M., Kari C., Rodeck U. (2000). The EGF receptor—An essential regulator of multiple epidermal functions. Eur. J. Dermatol..

[40] Flavahan W.A., Gaskell E., Bernstein B.E. (2017). Epigenetic plasticity and the hallmarks of cancer. Science.

[41] Al Aboud N.M., Tupper C., Jialal I. (2022). Genetics, Epigenetic Mechanism. StatPearls.

[42] Handy D.E., Castro R., Loscalzo J. (2011). Epigenetic modifications: Basic mechanisms and role in cardiovascular disease. Circulation.

[43] Penta D., Somashekar B.S., Meeran S.M. (2018). Epigenetics of skin cancer: Interventions by selected bioactive phytochemicals. Photodermatol. Photoimmunol. Photomed..

[44] Baylin S.B., Jones P.A. (2016). Epigenetic Determinants of Cancer. Cold Spring Harb. Perspect. Biol..

[45] Locke W.J., Guanzon D., Ma C., Liew Y.J., Duesing K.R., Fung K.Y.C., Ross J.P. (2019). DNA Methylation Cancer Biomarkers: Translation to the Clinic. Front. Genet..

[46] Nishiyama A., Nakanishi M. (2021). Navigating the DNA methylation landscape of cancer. Trends Genet..

[47] Strub T., Ballotti R., Bertolotto C. (2020). The “ART” of Epigenetics in Melanoma: From histone “Alterations, to Resistance and Therapies”. Theranostics.

[48] Hervas-Marin D., Higgins F., Sanmartin O., Lopez-Guerrero J.A., Bano M.C., Igual J.C., Quilis I., Sandoval J. (2019). Genome wide DNA methylation profiling identifies specific epigenetic features in high-risk cutaneous squamous cell carcinoma. PLoS ONE.

[49] Ehrlich M., Lacey M. (2013). DNA hypomethylation and hemimethylation in cancer. Adv. Exp. Med. Biol..

[50] Kim H.Y., Lee D.H., Shin M.H., Shin H.S., Kim M.K., Chung J.H. (2018). UV-induced DNA methyltransferase 1 promotes hypermethylation of tissue inhibitor of metalloproteinase 2 in the human skin. J. Dermatol. Sci..

[51] Pacella G., Capell B.C. (2021). Epigenetic and metabolic interplay in cutaneous squamous cell carcinoma. Exp. Dermatol..

[52] Lohcharoenkal W., Harada M., Loven J., Meisgen F., Landen N.X., Zhang L., Lapins J., Mahapatra K.D., Shi H., Nissinen L. (2016). MicroRNA-203 Inversely Correlates with Differentiation Grade, Targets c-MYC, and Functions as a Tumor Suppressor in cSCC. J. Investig. Dermatol..

[53] Lohcharoenkal W., Li C., Das Mahapatra K., Lapins J., Homey B., Sonkoly E., Pivarcsi A. (2021). MiR-130a Acts as a Tumor Suppressor MicroRNA in Cutaneous Squamous Cell Carcinoma and Regulates the Activity of the BMP/SMAD Pathway by Suppressing ACVR1. J. Investig. Dermatol..

[54] Zhang J., Cao Z., Yang G., You L., Zhang T., Zhao Y. (2019). MicroRNA-27a (miR-27a) in Solid Tumors: A Review Based on Mechanisms and Clinical Observations. Front. Oncol..

[55] Garcia-Sancha N., Corchado-Cobos R., Perez-Losada J., Canueto J. (2019). MicroRNA Dysregulation in Cutaneous Squamous Cell Carcinoma. Int. J. Mol. Sci..

[56] Yin S., Lin X. (2021). MicroRNA-21 Contributes to Cutaneous Squamous Cell Carcinoma Progression via Mediating TIMP3/PI3K/AKT Signaling Axis. Int. J. Gen. Med..

[57] Wang J., Lin Y., Jiang T., Gao C., Wang D., Wang X., Wei Y., Liu T., Zhu L., Wang P. (2019). Up-regulation of TIMP-3 and RECK decrease the invasion and metastasis ability of colon cancer. Arab. J. Gastroenterol..

[58] Tian J., Shen R., Yan Y., Deng L. (2018). miR-186 promotes tumor growth in cutaneous squamous cell carcinoma by inhibiting apoptotic protease activating factor-1. Exp. Ther. Med..

[59] Canueto J., Cardenoso-Alvarez E., Garcia-Hernandez J.L., Galindo-Villardon P., Vicente-Galindo P., Vicente-Villardon J.L., Alonso-Lopez D., De Las Rivas J., Valero J., Moyano-Sanz E. (2017). MicroRNA (miR)-203 and miR-205 expression patterns identify subgroups of prognosis in cutaneous squamous cell carcinoma. Br. J. Dermatol..

[60] Mulvaney P.M., Schmults C.D. (2020). Molecular prediction of metastasis in cutaneous squamous cell carcinoma. Curr. Opin. Oncol..

[61] Gillespie J., Skeeles L.E., Allain D.C., Kent M.N., Peters S.B., Nagarajan P., Yu L., Teknos T.N., Olencki T., Toland A.E. (2016). MicroRNA expression profiling in metastatic cutaneous squamous cell carcinoma. J. Eur. Acad. Dermatol. Venereol..

[62] Hernandez-Ruiz E., Toll A., Garcia-Diez I., Andrades E., Ferrandiz-Pulido C., Masferrer E., Yebenes M., Jaka A., Gimeno J., Gimeno R. (2018). The Polycomb proteins RING1B and EZH2 repress the tumoral pro-inflammatory function in metastasizing primary cutaneous squamous cell carcinoma. Carcinogenesis.

[63] Chiles M.C., Ai L., Zuo C., Fan C.Y., Smoller B.R. (2003). E-cadherin promoter hypermethylation in preneoplastic and neoplastic skin lesions. Mod. Pathol..

[64] Murao K., Kubo Y., Ohtani N., Hara E., Arase S. (2006). Epigenetic abnormalities in cutaneous squamous cell carcinomas: Frequent inactivation of the RB1/p16 and p53 pathways. Br. J. Dermatol..

[65] Takeuchi T., Liang S.B., Matsuyoshi N., Zhou S., Miyachi Y., Sonobe H., Ohtsuki Y. (2002). Loss of T-cadherin (CDH13, H-cadherin) expression in cutaneous squamous cell carcinoma. Lab. Investig..

[66] Li L., Jiang M., Feng Q., Kiviat N.B., Stern J.E., Hawes S., Cherne S., Lu H. (2015). Aberrant Methylation Changes Detected in Cutaneous Squamous Cell Carcinoma of Immunocompetent Individuals. Cell Biochem. Biophys..

[67] Liang J., Kang X., Halifu Y., Zeng X., Jin T., Zhang M., Luo D., Ding Y., Zhou Y., Yakeya B. (2015). Secreted frizzled-related protein promotors are hypermethylated in cutaneous squamous carcinoma compared with normal epidermis. BMC Cancer.

[68] Darr O.A., Colacino J.A., Tang A.L., McHugh J.B., Bellile E.L., Bradford C.R., Prince M.P., Chepeha D.B., Rozek L.S., Moyer J.S. (2015). Epigenetic alterations in metastatic cutaneous carcinoma. Head Neck.

[69] Bonnet D., Dick J.E. (1997). Human acute myeloid leukemia is organized as a hierarchy that originates from a primitive hematopoietic cell. Nat. Med..

[70] O’Connor M.L., Xiang D., Shigdar S., Macdonald J., Li Y., Wang T., Pu C., Wang Z., Qiao L., Duan W. (2014). Cancer stem cells: A contentious hypothesis now moving forward. Cancer Lett..

[71] Polyak K., Haviv I., Campbell I.G. (2009). Co-evolution of tumor cells and their microenvironment. Trends Genet..

[72] Rycaj K., Tang D.G. (2015). Cell-of-Origin of Cancer versus Cancer Stem Cells: Assays and Interpretations. Cancer Res..

[73] Beck B., Blanpain C. (2013). Unravelling cancer stem cell potential. Nat. Rev. Cancer.

[74] Greaves M., Maley C.C. (2012). Clonal evolution in cancer. Nature.

[75] Nassar D., Blanpain C. (2016). Cancer Stem Cells: Basic Concepts and Therapeutic Implications. Annu. Rev. Pathol..

[76] Plaks V., Kong N., Werb Z. (2015). The cancer stem cell niche: How essential is the niche in regulating stemness of tumor cells?. Cell Stem Cell.

[77] Cojoc M., Mabert K., Muders M.H., Dubrovska A. (2015). A role for cancer stem cells in therapy resistance: Cellular and molecular mechanisms. Semin. Cancer Biol..

[78] Jian Z., Strait A., Jimeno A., Wang X.J. (2017). Cancer Stem Cells in Squamous Cell Carcinoma. J. Invest. Dermatol..

[79] Huang Z., Wu T., Liu A.Y., Ouyang G. (2015). Differentiation and transdifferentiation potentials of cancer stem cells. Oncotarget.

[80] Gupta P.B., Pastushenko I., Skibinski A., Blanpain C., Kuperwasser C. (2019). Phenotypic Plasticity: Driver of Cancer Initiation, Progression, and Therapy Resistance. Cell Stem Cell.

[81] Naz F., Shi M., Sajid S., Yang Z., Yu C. (2021). Cancer stem cells: A major culprit of intra-tumor heterogeneity. Am. J. Cancer Res..

[82] Iseghohi S.O. (2016). Cancer stem cells may contribute to the difficulty in treating cancer. Genes Dis..

[83] Prasetyanti P.R., Medema J.P. (2017). Intra-tumor heterogeneity from a cancer stem cell perspective. Mol. Cancer.

[84] Kumar V.E., Nambiar R., De Souza C., Nguyen A., Chien J., Lam K.S. (2022). Targeting Epigenetic Modifiers of Tumor Plasticity and Cancer Stem Cell Behavior. Cells.

[85] Liao G., Tang J., Bai J. (2023). Early development of esophageal squamous cell cancer: Stem cells, cellular origins and early clone evolution. Cancer Lett..

[86] Argenziano G., Fargnoli M.C., Fantini F., Gattoni M., Gualdi G., Pastore F., Pellacani G., Quaglino P., Queirolo P., Troiani T. (2022). Identifying candidates for immunotherapy with cemiplimab to treat advanced cutaneous squamous cell carcinoma: An expert opinion. Ther. Adv. Med. Oncol..

[87] Stratigos A.J., Garbe C., Dessinioti C., Lebbe C., Bataille V., Bastholt L., Dreno B., Concetta Fargnoli M., Forsea A.M., Frenard C. (2020). European interdisciplinary guideline on invasive squamous cell carcinoma of the skin: Part 2. Treatment. Eur. J. Cancer.

[88] Garcia-Foncillas J., Tejera-Vaquerizo A., Sanmartin O., Rojo F., Mestre J., Martin S., Azinovic I., Mesia R. (2022). Update on Management Recommendations for Advanced Cutaneous Squamous Cell Carcinoma. Cancers.

[89] Ferreira L.O., Cernea S.S., Weber M.B., Ferreira I.G., Sil A.B. (2021). Advanced squamous cell carcinoma and immunotherapy: New therapeutic perspectives. Surg. Cosmet. Dermatol..

[90] Stratigos A.J., Garbe C., Dessinioti C., Lebbe C., Bataille V., Bastholt L., Dreno B., Fargnoli M.C., Forsea A.M., Frenard C. (2020). European interdisciplinary guideline on invasive squamous cell carcinoma of the skin: Part 1. epidemiology, diagnostics and prevention. Eur. J. Cancer.

[91] Stanganelli I., Spagnolo F., Argenziano G., Ascierto P.A., Bassetto F., Bossi P., Donato V., Massi D., Massone C., Patuzzo R. (2022). The Multidisciplinary Management of Cutaneous Squamous Cell Carcinoma: A Comprehensive Review and Clinical Recommendations by a Panel of Experts. Cancers.

[92] National Comprehensive Cancer Network Squamous Cell Skin Cancer. https://www.nccn.org/professionals/physician_gls/pdf/squamous.pdf.

[93] Yanagi T., Kitamura S., Hata H. (2018). Novel Therapeutic Targets in Cutaneous Squamous Cell Carcinoma. Front. Oncol..

[94] Maubec E. (2020). Update on the Management of Cutaneous Squamous Cell Carcinoma. Acta Derm. Venereol..

[95] Alberti A., Bossi P. (2021). Immunotherapy for Cutaneous Squamous Cell Carcinoma: Results and Perspectives. Front. Oncol..

[96] Garcia-Sancha N., Corchado-Cobos R., Bellido-Hernandez L., Roman-Curto C., Cardenoso-Alvarez E., Perez-Losada J., Orfao A., Canueto J. (2021). Overcoming Resistance to Immunotherapy in Advanced Cutaneous Squamous Cell Carcinoma. Cancers.

[97] Villegas-Romero I., Jiménez-Gallo D., Gutiérrez-Bayard L., Linares-Barrios M. (2021). Carcinoma epidermoide cutáneo avanzado tratado con pembrolizumab. Actas Dermo-Sifiliogr.

[98] Peris K., Piccerillo A., Del Regno L., Di Stefani A. (2022). Treatment approaches of advanced cutaneous squamous cell carcinoma. J. Eur. Acad. Dermatol. Venereol..

[99] Migden M.R., Rischin D., Schmults C.D., Guminski A., Hauschild A., Lewis K.D., Chung C.H., Hernandez-Aya L., Lim A.M., Chang A.L.S. (2018). PD-1 Blockade with Cemiplimab in Advanced Cutaneous Squamous-Cell Carcinoma. N. Engl. J. Med..

[100] Kozakiewicz P., Grzybowska-Szatkowska L. (2018). Application of molecular targeted therapies in the treatment of head and neck squamous cell carcinoma. Oncol. Lett..

[101] Grob J.J., Mendoza R.G., Basset-Seguin N., Vornicova O., Schachter J., Joshi A., Meyer N., Grange F., Piulats J.M., Bauman J. (2019). LBA72—Pembrolizumab for recurrent/metastatic cutaneous squamous cell carcinoma (cSCC): Efficacy and safety results from the phase II KEYNOTE-629 study. Ann. Oncol..

[102] Wessely A., Steeb T., Leiter U., Garbe C., Berking C., Heppt M.V. (2020). Immune Checkpoint Blockade in Advanced Cutaneous Squamous Cell Carcinoma: What Do We Currently Know in 2020?. Int. J. Mol. Sci..

[103] Miller D.M., Faulkner-Jones B.E., Stone J.R., Drews R.E. (2017). Complete pathologic response of metastatic cutaneous squamous cell carcinoma and allograft rejection after treatment with combination immune checkpoint blockade. JAAD Case Rep..

[104] Ferris R.L., Licitra L., Fayette J., Even C., Blumenschein G., Harrington K.J., Guigay J., Vokes E.E., Saba N.F., Haddad R. (2019). Nivolumab in Patients with Recurrent or Metastatic Squamous Cell Carcinoma of the Head and Neck: Efficacy and Safety in CheckMate 141 by Prior Cetuximab Use. Clin. Cancer Res..

[105] Montaudie H., Viotti J., Combemale P., Dutriaux C., Dupin N., Robert C., Mortier L., Kaphan R., Duval-Modeste A.B., Dalle S. (2020). Cetuximab is efficient and safe in patients with advanced cutaneous squamous cell carcinoma: A retrospective, multicentre study. Oncotarget.

[106] Perez M.C., Miura J.T., Naqvi S.M.H., Kim Y., Holstein A., Lee D., Sarnaik A.A., Zager J.S. (2018). Talimogene Laherparepvec (TVEC) for the Treatment of Advanced Melanoma: A Single-Institution Experience. Ann. Surg. Oncol..

[107] Middleton M., Aroldi F., Sacco J., Milhem M.M., Curti B.D., Vanderwalde A.M., Baum S., Samson A., Pavlick A.C., Chesney J.A. (2020). An open-label, single-arm, phase II clinical trial of RP1, an enhanced potency oncolytic herpes virus, combined with nivolumab in four solid tumor types: Initial results from the skin cancer cohorts. J. Clin. Oncol..

